# Reply to Schluessel et al. Comment on “Cancello et al. Sarcopenia Prevalence Among Hospitalized Patients with Severe Obesity: An Observational Study. *J. Clin. Med.* 2024, *13*, 2880”

**DOI:** 10.3390/jcm13226687

**Published:** 2024-11-07

**Authors:** Raffaella Cancello, Ettore Brenna, Davide Soranna, Antonella Zambon, Valentina Villa, Gianluca Castelnuovo, Lorenzo Maria Donini, Luca Busetto, Paolo Capodaglio, Amelia Brunani

**Affiliations:** 1 Obesity Unit, Laboratory of Nutrition and Obesity Research, Department of Endocrine and Metabolic Diseases, IRCCS Istituto Auxologico Italiano, 20145 Milan, Italy; r.cancello@auxologico.it; 2 Biostatistic Unit, IRCCS Istituto Auxologico Italiano, 20145 Milan, Italy; e.brenna@auxologico.it (E.B.); d.soranna@auxologico.it (D.S.); antonella.zambon@unimib.it (A.Z.); 3 Department of Statistics and Quantitative Methods, University of Milan-Bicocca, 20145 Milan, Italy; 4 Psychology Research Laboratory, IRCCS Istituto Auxologico Italiano, 20145 Milan, Italy; v.villa@auxologico.it (V.V.); gianluca.castelnuovo@unicatt.it (G.C.); 5 Department of Psychology, Catholic University of Milan, 20145 Milan, Italy; 6 Department of Experimental Medicine, Sapienza University, 00185 Rome, Italy; lorenzomaria.donini@uniroma.it; 7 Department of Medicine, University of Padova, 35100 Padova, Italy; luca.busetto@unipd.it; 8 Department of Surgical Sciences, Physical Medicine and Rehabilitation, University of Turin, 10126 Turin, Italy; p.capodaglio@auxologico.it; 9 Laboratory of Biomechanics, Rehabilitation and Ergonomics, IRCCS Istituto Auxologico Italiano, Piancavallo, 28044 Verbania, Italy

We appreciate the interest of Schluessel S. et al. [1] regarding our recently published study on sarcopenic obesity (SO) [2]. Their comments give us the opportunity to clarify and expand the issue about the consideration of fat mass (FM) percentage. We are aware that the new ESPEN/EASO guidelines for SO diagnosis [3,4] recommend considering both impaired skeletal muscle function and muscle deficit, along with the presence of fat mass percentage (FM%) excess, using sex-, ethnicity-, and age-specific cut-offs. In our study, all patients were hospitalized for obesity and had, as reported, at least one comorbid condition. The cohort was entirely composed of Caucasian inpatients, and 59% of the participants had class III obesity (BMI ≥ 40 kg/m^2^). It is crucial to note that this cohort represents a population with extreme FM excess rather than common forms of obesity. Hence, the FM% was included in the diagnostic criteria, and no participants were excluded due to FM% deficiency, as detailed in Figure 1. Additionally, as shown in Table 1, and considering the cut-offs for sex and age recommended for the Caucasian population [3,4,5], none of the patients exhibited FM% below the suggested thresholds for SO diagnosis. The BMI was notably high across all age groups, indicating that obesity may be a significant risk factor for sarcopenia, also in non-geriatric populations. In each age range, FM% exceeded the suggested cut-offs for SO diagnosis (i.e., age 20–39 years: >39% for females, F, >26% for males, M; age 40–59 years: >41% for F, >29% for M; age 60–79 years: >43% for F, >31% for M) reported for Caucasians [3,4,5].

Although a portion of our cohort consisted of geriatric patients, the results are not restricted to this age group and are not directly comparable to studies focusing solely on geriatric populations. Our analysis was centered on individuals with extreme obesity and FM% excess, spanning various age groups in both sexes.

We trust that this clarification, along with the additional data on FM%, addresses the concerns raised and reinforces the robustness of our diagnostic approach for SO diagnosis applying ESPEN/EASO-SO diagnostic criteria [3,4].

We are grateful for the opportunity to engage in this important discussion and remain committed to advancing the understanding and clinical assessment of sarcopenic obesity.

## Figures and Tables

**Figure 1:**
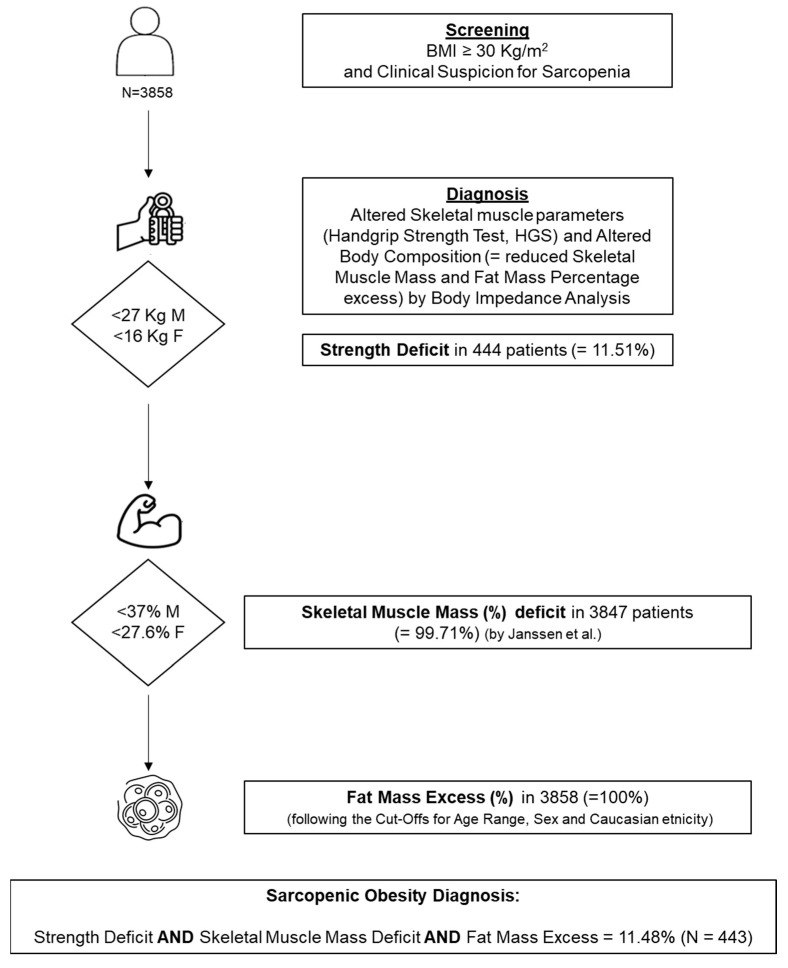
Flowchart of ESPEN/EASO-SO diagnostic criteria application in the whole cohort with the corresponding number and percentage of patients satisfying the diagnostic criteria [3,4,5].

**Table 1:** Sarcopenic obesity (SO), percentage of fat mass (FM, %) and body mass index (BMI) of the studied cohort [2] reported by sex (female and male) and by age range for Caucasian population [3,4,5]. Data are reported as mean ± standard deviation (SD).

	**Female (N = 2348)**			
**Age Range, Years (Patient Number, N)**	**18–39 (N = 253)**	**40–59 (N = 1058)**	**60–79 (N = 958)**	**≥80 (N = 79)**
BMI (Kg/m^2^), mean ± SD	43.98 ± 6.71	43.74 ± 6.94	43.08 ± 6.06	40.50 ± 5.50
SO, number of patients, N, (%)	8 (3%)	89 (8%)	169 (18%)	25 (32%)
FM (%) mean ± SD, (all)	52.81 ± 4.11	51.36 ± 4.51	52.99 ± 4.55	50.93 ± 4.39
FM (%) mean ± SD, SO patients	54.35 ± 3.17	51.20 ± 5.57	52.60 ± 4.21	50.76 ± 4.69
FM (%) mean ± SD, no-SO patients	52.76 ± 4.13	51.37 ± 4.41	53.08 ± 4.62	51.01 ± 4.29
	**Male (N = 1510)**			
**Age Range, Years (Patient Number, N)**	**18–39 (N = 193)**	**40–59 (N = 727)**	**60–79 (N = 562)**	**≥80 (N = 28)**
BMI (Kg/m^2^), mean ± SD	44.81 ± 7.47	43.69 ± 7.21	41.11 ± 5.61	38.3 ± 3.87
SO, number of patients, N, (%)	6 (3%)	49 (7%)	88 (16%)	9 (32%)
FM (%) mean ± SD, (all)	44.30 ± 5.18	41.27 ± 5.82	42.77 ± 5.31	41.11 ± 4.39
FM (%) mean ± SD, SO patients	46.75 ± 4.38	40.95 ± 5.92	42.68 ± 6.23	41.12 ± 3.45
FM (%) mean ± SD, no-SO patients	44.22 ± 5.19	41.29 ± 5.82	42.79 ± 5.13	41.11 ± 4.85
